# Vitamin D is associated with metabotropic but not neurotrophic effects of exercise in ovariectomized rats

**DOI:** 10.1186/s13098-017-0288-z

**Published:** 2017-11-15

**Authors:** Parvin Babaei, Samaneh Ghorbani Shirkouhi, Rastegar Hosseini, Bahram Soltani Tehrani

**Affiliations:** 1 Cellular & Molecular Research Center, Faculty of Medicine, Guilan University of Medical Sciences, Rasht, 2263 Iran; 2 Department of Physiology, Faculty of Medicine, Guilan University of Medical Sciences, Rasht, Iran; 3 Neuroscience Research Center, Guilan University of Medical Sciences, Rasht, Iran; 4 Department of Physical Education and Sport Sciences, Kermanshah Branch, Islamic Azad University, Kermanshah, Iran; 5 Department of Pharmacology, Faculty of Medicine, Guilan University of Medical Sciences, Rasht, Iran

**Keywords:** Metabolic syndrome, Memory, Vitamin D, BDNF, Irisin, Aerobic exercise

## Abstract

**Purpose:**

Here, we studied the beneficial effects of aerobic exercise on metabolic syndrome components, cognitive performance, brain derived neurotrophic factor (BDNF) and irisin in ovariectomized rats with different serum vitamin D (Vit D) status.

**Methods:**

Eighty female wistar rats were divided into 2 groups of sham operated (sham, n = 8), and ovariectomized (OVX, n = 72). Then OVX were divided into 9 groups of receiving combination of exercise protocol with low dose of Vit D (OVX + EXE + LD), high dose of Vit D (OVX + EXE + HD), Vit D deficiency (OVX + EXE − D), and (OVX + EXE + Veh). Also non exercised groups of OVX receiving high dose of Vit D (OVX + HD), low dose of Vit D (OVX + LD), Vit D deficiency (OVX − D), and Veh (OVX + Veh) were included. After 2 months of related interventions, spatial memory was assessed using Morris water maze (MWM), and then metabolic syndrome components were measured.

**Results:**

High dose of Vit D supplementation showed significant reduction in weight (p = 0.001), lipid profiles (p = 0.001), visceral fat (p = 0.001) and waist circumference (p = 0.001) regardless of exercising or not, with no change in cognitiive function. Serum BDNF level was significantly higher in Vit D deficient group (p = 0.001), and was decreased in the OVX + HD. In contrary, irisin did not show any significant relationship with serum concentration of Vit D, while it was significantly elevated in the exercised groups compared with non-exercised counterparts.

**Conclusion:**

Vit D insufficiency deteriorates metabolic syndrome components, and elevates serum BDNF as a compensatory metabotropic factor, and further supplementation significantly attenuates these components parallel with reduction in BDNF. In addition, aerobic exercise successfully induces various metabolic benefits, provided optimum serum level of Vit D.

## Background

Metabolic syndrome (MetS) is a clinical challenge worldwide due to obesity, and sedentary life habits. Based on the previous studies, individuals with three criteria (central obesity, high triglycerides, low high-density lipoprotein cholesterol, hypertension, and hyperglycemia) are diagnosed with MetS [1–3]. During menopausal period, estrogen withdrawal predisposes women to cardiovascular [4] and cognitive dysfunctions [5].

On the other hand, chronic low serum Vit D concentration in the elderly women is an important public health concern due to its high prevalence of 50–80% [6].

It has been known that insufficient serum Vit D_3_ alters metabolite function [7], causes insulin resistance [8, 9], and develops MetS components [3, 10]. Also Vit D insufficiency may leads to develop dementias, including Alzheimer’s disease [11] and cognitive impairment [12]. Considering the fact that Vit D receptors (VDR) are located in the human cortex and hippocampus, which are key areas for cognition [13], Vit D is assumed to be important in learning and memory.

One of the non-pharmacological tools to prevent both metabolic syndrome and cognitive deficit is aerobic exercise [14]. Some of the beneficial effects of aerobic exercise take place via elevation in serum BDNF [15, 16] and irisin [17]. It is postulated that exercised skeletal muscles secrete a myokine, named irisin, which passes blood brain barrier and secrets BDNF [14, 18, 19]. BDNF not only boosts learning and memory [14, 20, 21], as a neurotrophic factor [22, 23], but also it recently stands as a metabotropic factor too [24, 25]. It has not been understood why some of the obese individuals do not show any significant metabolic [26, 27] or cognitive [28] improvements after physical activities. Therefore, the present study was designed to clarify whether or not Vit D insufficiency might be the reason to prevent exercise beneficial effects? Thus, the objective of this study was co-treatment of Vit D supplementation with aerobic exercise on cognitive performance, metabolic syndrome components, serum BDNF and irisin level. To approach these objectives we used ovariectomized rats as a model of menopause and metabolic syndrome [29, 30].

## Methods

### Animals

Female rats (3 months age and 180–200 g weight) were housed four per cage and all rats (except Vit D deficient group) fed standard-pellet rat chow (Table 1) and tap water ad libitum. Room temperature was maintained at 22 ± 2 °C with a 12/12 h light/dark cycle (light on 7:00A.M.). All experiments were performed in accordance with National Institutes of Health guide for the care and use of laboratory animals (NIH Publications No. 8023, revised 1978) modified by ethical committee of Guilan University of medical sciences, Rasht, Iran, IR.GUMS.REC.1394.54.

**Table 1:** Ingredients of Rodents standard diet per 100 g

Protein (g)	Fat (g)	Fiber (g)	Ashes (g)	Calcium (g)	Phosphorus (g)	Salt (g)	Moisture (g)	Energy (kcal)	Vitamin D (IU)
22.5–23.5	3.5	4–5	10	0.95–1	0.65–0.7	0.5–0.55	10	2850–2900	5000

### Ovariectomy surgery

Eighty female wistar rats were divided into 2 groups of sham operated (sham, n = 8), and ovariectomized (OVX, n = 72). Then OVX were divided into 9 groups of receiving combination of exercise protocol with low dose of Vit D (OVX + EXE + LD), high dose of Vit D (OVX + EXE + HD), Vit D deficiency (OVX + EXE − D), and Veh (OVX + EXE + Veh). Also OVX receiving high dose of Vit D (OVX + HD), low dose of Vit D (OVX + LD), Vit D deficiency (OVX − D), and Veh (OVX + Veh) were included. Rats were ovariectomized and sham-operated under general anesthesia with an intraperitoneal injection of 75 mg/kg ketamine (50 mg/ml, TRITTAU, Germany) and 5 mg/kg of xylazine (20 mg/ml, SciENcelab, Hoston) and ovaries were removed by one midline incision on the abdomen, then animals left for recovery. Three weeks after the surgery, animals displayed weight gain, dyslipidemia, high abdominal obesity and visceral fat.

### Vit D supplementation

Based on the ingredients of animal’s food given by manufacture and subtracting consumed food from the total given per cage (Table 1), each animal of the LD group received 100 IU/kg/week Vit D (from food source, Tables 1, 2). HD group received 10,000 IU/kg/week Vit D [1000 IU/kg/week from food source and 9000 (10,000–1000) IU from injection]. Animals received Vit D_3_ (drug factory of Abureyhan Birooni, Tehran, Iran) injection (s.c) once a week, and control group received sesame oil as vehicle of Vit D. To balance the diet on isocaloric regimen, Vit D deficient and low Vit D groups received wheat-based food based on nutrition software prepared in our lab [31]. The ingredients of this Vit D free diet is displayed in Table 2.

**Table 2:** Ingredients of Vit D free diet per 100g

Energy (Kcal)	Fat (g)	Saturated fat (g)	Sodium (mg)	Potassium (mg)	Carbohydrate (g)	Fiber (g)	Sugar (g)	Protein (g)	Vitamin A (IU)	Vitamin C (mg)	Calcium (mg)	Iron (mg)	Vitamin E (mg)
270	3.4	0.8	519	185	49.5	4.2	6.1	10.4	2	0.2	138	3.5	0.2

Thiamine (mg)	Riboflavin (mg)	Niacin (mg)	B6 (mg)	Folate (µg)	Pantothenic acid (mg)	Magnesium (mg)	Zinc (mg)	Selenium (µg)	Phosphorus (mg)	Copper (mg)	Manganese (mg)	Water (g)	Vitamin D
0.5	0.3	5.9	0.1	85	0.8	45	1.2	28.8	153	0.2	1.2	34.5	0

### Aerobic exercise training

Aerobic training included running on a rodent treadmill (Daneshsalar Iranian Co, Tehran, Iran) with an incremental pattern (from 12 min/day at 12 m/min, 0% slope, up to 40 min/day at 25 m/min, 10% slope for the last 8 weeks) (Table 3) [32]. The regular endurance exercise used in this study was equivalent to 70–85% VO_2max_ [33].

**Table 3:** Treadmill exercise schedule during the 8 weeks

	Adaptation	Week 1	Week 2	Week 3	Week 4	Week 5	Week 6	Week 7	Week 8
Velocity (m/min)	10	12	15	15	20	20	23	25	25
Duration (min)	10	12	15	20	20	30	38	40	40
Treadmill slope (degree)	0	0	0	5	5	7	7	10	10

### Behavioral test

Protocol used for Morris water maze (MWM) included 5 days, each day consisted of 1 block of 4 trials, and each trial 60 s with an interval of 20 min. MWM consisted of a large circular pool with 148 cm diameter, divided to four directions (north east, north west, south east, south west), containing water at around 22 °C. A hidden platform with 10 cm diameter was kept 1.5 cm below the water surface in the target quadrant. A video camera was placed above the center of the pool to capture images of the swimming animal, and this connected to a video recorder [34, 35] and “Ethovision 7 Noldus” software. Times spent in the target quadrant and reach to the hidden platform as well as swimming speed were considered.

### Morphometric and chemical assays

At the end of the behavioral test, animals were weighed and their waist circumference was measured on the largest zone of the rat abdomen using a centimeter [36].

Then animals were sacrificed by chloroform and blood was collected from the inferior vena cava by a syringe into the EDTA containing tubes. Finally, bloods were centrifuged for 15 min at 3000 rpm, and stored at – 80 °C for further analysis.

Visceral fat were completely removed from mesenteric, urogenital and retroperitoneal and weighed immediately to avoid evaporation using a weighing-scale (Doulton). The mesenteric fat included the adipose tissue surrounding the gastrointestinal tract. The urogenital fat included the adipose tissue surrounding the kidneys, ureters and bladder as well as ovaries, oviducts and uterus. The retroperitoneal fat consisted of the distinct deposit behind each kidney along the lumbar muscles.

Serum Vit D and BDNF were assessed using the ELISA (Immuno Diagnostics System Ltd., Boldon, UK) and (Bosterbio, Picokine, Canada) respectively. Irisin was assessed using ELISA (Zellbio Kit, GmbH, Germany) and lipids profile were measured using enzymatic analysis kits (Asan Pharmaceuticals, Hwasung, Korea).

### Statistical analysis

Normality of variables was evaluated by Shapiro–Wilk test, then repeated measure and ANOVA with post hoc Tukey test were used for acquisition and memory retrieval respectively. Kruscall–Wallis and Bonferroni post hoc were used to compare between groups differences of nonparametric variables (BDNF, BMI, Ca, Visceral fat, Waist circumference). HDL, nonHDL, TC, TG, Weight, Irisin and VitD were analyzed using ANOVA followed by post hoc Tukey test. p value < 0.05 were considered statistically significant, and results are expressed as the mean ± SEM.

## Results

Three weeks after ovariectomy, animals showed an increase in body weight BMI (p = 0.001), visceral fat (p = 0.018) and waist circumference (p = 0.001) compared with SHAM group (Table 4). There was a statistically significant between groups difference in Vit D [F(9,70) = 772.15, p = 0.001]. The highest level of Vit D was detected in OVX + EXE + HD (117.04 ± 1.43 nmol/l, p = 0.001) and OVX + HD (107.06 ± 0.45 nmol/l, p = 0.001), but the lowest one in OVX − D (65.36 ± 0.92 nmol/l, p = 0.001) and OVX + EXE − D (67.13 ± 1.07 nmol/l, p = 0.001) groups.

**Table 4:** The morphometric and hormonal variables after 8-week experimental period

Variables	Weight (g)	BMI (g/cm^2^)	Visceral fat (g)	Waist circumference (cm)	Vitamin D (nmol/l)	Calcium (mg/dl)
Groups						
OVX + EXE + HD	**233.45 ± 0.61	**0.54 ± 0.001	**8.29 ± 0.050	**10.75 ± 0.231	**117.04 ± 1.43	8.9 ± 1.32
OVX + EXE + LD	245.14 ± 0.26	0.574 ± 0.009	9.20 ± 0.029	12.875 ± 0.26	102.1 ± 1.12	***7.96 ± 1.04
OVX + EXE − D	**259.25 ± 2.22	**0.57 ± 0.011	**15.43 ± 1.29	**19 ± 0	**67.13 ± 1.07	8.84 ± 0.92
OVX + EXE + Veh	271.61 ± 0.59	0.64 ± 0.001	9.54 ± 0.024	13.76 ± 0.451	90.35 ± 1.28	9.42 ± 0.24
OVX + HD	**240.73 ± .048	**0.56 ± 0.001	**8.76 ± 0.009	**11.89 ± 0.27	**107.09 ± 1.27	10.11 ± 0.75
OVX + LD	267.69 ± 040	0.63 ± 0.001	9.46 ± 0.008	13.59 ± 0.46	93.66 ± 0.93	*9.06 ± 0.47
OVX − D	**273.5 ± 4.08	**0.64 ± 0.03	**19.25 ± 2.72	**20.38 ± 0.71	**65.36 ± 0.92	8.76 ± 1.61
OVX + Veh	275.44 ± 1.22	0.65 ± 0.003	9.94 ± 0.015	14.34 ± 0.20	83.43 ± 1.57	9.23 ± 0.62
OVX	^##^247.86 ± 0.31	^##^0.61 ± 0.001	^##^10.5 ± 0.42	^#^12.66 ± 0.48	^##^82.31 ± 0.37	^##^8.5 ± 0.8
Sham	214.7 ± 0.65	0.53 ± 0.002	9.2 ± 0.13	9.13 ± 0.2	83.63 ± 0.45	9.19 ± 0.39

OVX + EXE + HD: ovariectomy + aerobic training + high dose of Vit D, OVX + EXE + LD: ovariectomy + aerobic training + low dose of Vit D, OVX + EXE − D: ovariectomy + aerobic training + Vit D deficiency, OVX + EXE + Veh: ovariectomy + aerobic training + sesame oil, OVX + HD: ovariectomy + low dose of VitD, OVX + LD, OVX − D: ovariectomy + VitD deficiency, OVX + Veh: ovariectomy + sesame oil, sham: sham-operated. OVX: ovariectomy. OVX and SHAM group values are about 3 weeks after ovariectomy surgery. **p = 0.001, compared with Veh group. ^#^p = 0.01, compared with SHAM. ^##^p = 0.001, compared with SHAM. *p = 0.046, compared with Veh. ***p = 0.003, compared with Veh. Values are expressed as mean + SE, n = 8 rats per group

BMI showed statistically significant between groups difference as determined by ANOVA [F(9,70) = 423.54, p = 0.001]. The group of OVX + HD compared with OVX − D showed significant reduction in both weight (− 3.16% vs. 15.4%) and BMI (− 9.8% vs. 8.5%) (p = 0.001). In addition, OVX + EXE + HD showed significant reduction in weight (− 5.9% vs. 11.3%) and BMI (− 13.11% vs. − 6.5%, p = 0.003) compared with OVX + EXE − D (Table 4).

There was a statistically significant between groups difference in waist circumference [F(9,70) = 84.72, p = 0.001] and visceral fat [F(9,70) = 13.94, p = 0.001]. Visceral fat in OVX + HD and OVX + EXE + HD weighed 8.75 g compared with 19.25 g in OVX − D and 15.43 g in OVX + EXE − D (Table 4).

**Fig. 1:**
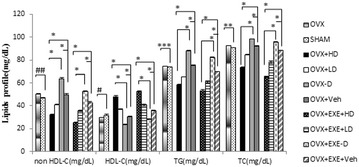
Comparison of mean + Se lipids profile among different groups. OVX + EXE + HD: ovariectomy + aerobic training + high dose of Vit D, OVX + EXE + LD: ovariectomy + aerobic training + low dose of Vit D, OVX + EXE − D: ovariectomy + aerobic training + Vit D deficiency, OVX + EXE + Veh: ovariectomy + aerobic training + sesame oil, OVX + HD: ovariectomy + low dose of Vit D, OVX + LD, OVX − D: ovariectomy + VitD deficiency, OVX + Veh: ovariectomy + sesame oil, sham: sham-operated. OVX: ovariectomy. n = 8 rats per group. *p = 0.001, compared with Veh group, ^##^p = 0.001, **p = 0.002, ***p = 0.01, ^#^p = 0.03, compared with sham group

Also statistically significant reduction was found in HDL [F(9,70) = 208.84), (p = 0.001)] LDL [F(9,70) = 128.68), (p = 0.001)], TG [F(9,70) = 358.29), (p = 0.001)] and TC [F(9,70) = 394.02), (p = 0.001)]. OVX + HD and OVX + EXE + HD showed elevation in serum HDL (p = 0.01), but reduction in non HDL and TG compared with OVX − D (p = 0.001, Fig. 1).

### Behavioral results

**Fig. 2:**
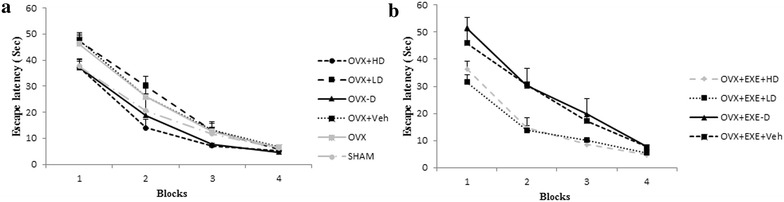
**a** Comparison of escape latency in 4 blocks among OVX, sham, OVX + HD, OVX + LD, OVX − D and OVX + Veh groups. **b** Comparison of escape latency in acquisition phase of spatial memory in MWM among OVX + EXE + HD, OVX + EXE + LD, OVX + EXE − D and OVX + EXE + Veh groups. OVX + EXE + HD: ovariectomy + aerobic training + high dose of Vit D, OVX + EXE + LD: ovariectomy + aerobic training + low dose of Vit D, OVX + EXE − D: ovariectomy + aerobic training + Vit D deficiency, OVX + EXE + Veh: ovariectomy + aerobic training + sesame oil, OVX + HD: ovariectomy + low dose of VitD, OVX + LD, OVX − D: ovariectomy + VitD deficiency, OVX + Veh: ovariectomy + sesame oil, sham: sham-operated. OVX: ovariectomy. Values are expressed as mean ± SEM, n = 8 rats per group

Escape latency time measured in MWM in the 2nd, 3rd and 4th days of all groups showed trends of reduction (Fig. 2a, b) indicating that all animals learned the MWM task successfully. Since none of the groups differed in swimming speed [F(10,70) = 1.31, (p = 0.24)], the latency time to find the platform and total time spent in the target quadrant (TTS) were used here, as indicators of learning performance.

**Fig. 3:**
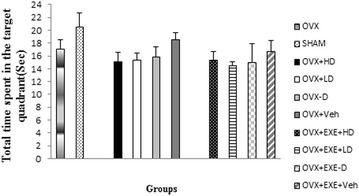
Comparison of total time spent in the target quadrant in retrieval phase of spatial memory in MWM among different groups. OVX + EXE + HD: ovariectomy + aerobic training + high dose of Vit D, OVX + EXE + LD: ovariectomy + aerobic training + low dose of Vit D, OVX + EXE − D: ovariectomy + aerobic training + Vit D deficiency, OVX + EXE + Veh: ovariectomy + aerobic training + sesame oil, OVX + HD: Ovariectomy + low dose of VitD, OVX + LD, OVX − D: ovariectomy + VitD deficiency, OVX + Veh: ovariectomy + sesame oil, sham: sham-operated. OVX: ovariectomy. Values are expressed as mean + Se, n = 8 rats per group

**Fig. 4:**
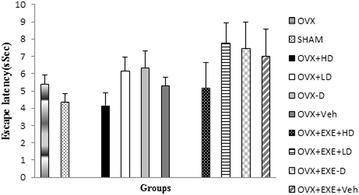
Comparison of escape latency in retrieval phase of spatial memory in MWM among different groups. OVX + EXE + HD: ovariectomy + aerobic training + high dose of Vit D, OVX + EXE + LD: ovariectomy + aerobic training + low dose of Vit D, OVX + EXE − D: ovariectomy + aerobic training + Vit D deficiency, OVX + EXE + Veh: ovariectomy + aerobic training + sesame oil, OVX + HD: ovariectomy + low dose of VitD, OVX + LD, OVX − D: ovariectomy + VitD deficiency, OVX + Veh: ovariectomy + sesame oil, sham: sham-operated. OVX: ovariectomy. Values are expressed as mean + Se, n = 8 rats per group

**Fig. 5:**
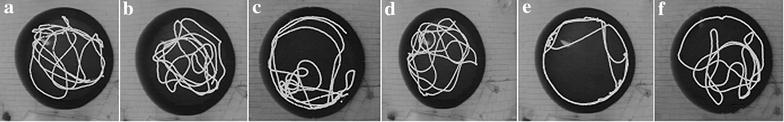
Ethovision tracking from retrieval phase in MWM. **a** OVX + EXE + HD, **b** OVX + EXE + LD, **c** OVX + EXE − D, **d** OVX + HD, **e** OVX + LD, **f** OVX − D

The OVX group displayed insignificant poor performance in acquisition (p = 1.000) and retrieval of memory (p = 0.240) compared with SHAM group. Vit D insufficiency had no significant effect neither on acquisition nor retrieval of memory compared with Veh group (Figs. 2a, 3 and 4). Also no significant change in TTS in the target quadrant in the OVX + EXE − D group was found compared with Veh group (14.95 ± 2.97 s vs. 16.7 ± 1.75 s, p = 1.0) (Fig. 3). As Figs. 2a, b, 3 and 4 show, Vit D supplementation for 8 weeks had no significant change on acquisition (p = 0.644, Fig. 2a) and retrieval of memory (p = 0.90, p = 0.17, Figs. 3, 4), compared with Veh group. Tukey’s post hoc test showed that the co-treatment of aerobic training with high dose of Vit D insignificantly decreased escape latency time compared with Veh group (5.2 ± 1.5 s vs. 7 ± 0.7 s, p = 0.083, Figs. 4, 5).

**Fig. 6:**
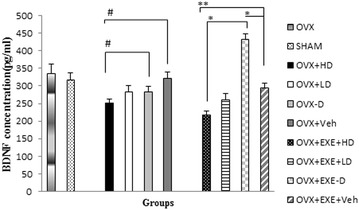
Comparison of serum BDNF concentration among different groups. OVX + EXE + HD: ovariectomy + aerobic training + high dose of Vit D, OVX + EXE + LD: ovariectomy + aerobic training + low dose of Vit D, OVX + EXE − D: ovariectomy + aerobic training + Vit D deficiency, OVX + EXE + Veh: ovariectomy + aerobic training + sesame oil, OVX + HD: ovariectomy + low dose of VitD, OVX + LD, OVX − D: ovariectomy + VitD deficiency, OVX + Veh: ovariectomy + sesame oil, sham: sham-operated. OVX: ovariectomy. Values are expressed as mean + Se, n = 8 rats per group. *p = 0.001, compared with OVX + EXE − D and Veh. **p = 0.003, compared with Veh. ^#^p = 0.046, compared with OVX-D and Veh

Statistically significant between groups difference was found in serum BDNF concentrations [F(9,70) = 10.57, (p = 0.001)]. Dunn-bonferroni post hoc test revealed the highest level of serum BDNF in the group with Vit D insufficiency (p = 0.001, Fig. 6). Co-treatment of aerobic training with high dose of Vit D, significantly decreased serum BDNF concentration compared with OVX + EXE + Veh (p = 0.003) and EXE − D (p = 0.001). Furthermore, significant negative correlation was found between Vit D and serum BDNF level (Fig. 6).

**Fig. 7:**
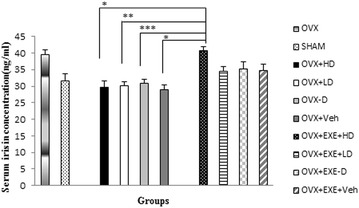
Comparison of serum irisin concentration among different groups. OVX + EXE + HD: ovariectomy + aerobic training + high dose of Vit D, OVX + EXE + LD: ovariectomy + aerobic training + low dose of Vit D, OVX + EXE − D: ovariectomy + aerobic training + Vit D deficiency, OVX + EXE + Veh: ovariectomy + aerobic training + sesame oil, OVX + HD: ovariectomy + low dose of VitD, OVX + LD, OVX − D: ovariectomy + VitD deficiency, OVX + Veh: ovariectomy + sesame oil, sham: sham-operated. OVX: ovariectomy. Values are expressed as mean + Se, n = 8 rats per group. *p = 0.001, **p = 0.002, ***p = 0.005 compared with OVX + EXE + HD

**Fig. 8:**
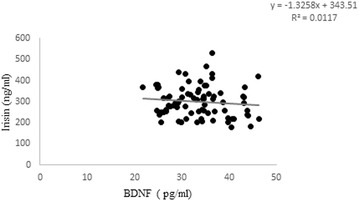
Correlation between serum concentration of BDNF and irisin. There was no significant correleation between BDNF and Irisin. p = 0.34, R^2^ = 0.0117, n = 8

There was a statistically significant between groups difference in serum irisin [F(9,70) = 5.62, (p = 0.001)]. Irisin did not show any significant change in groups supplemented with different doses of Vit D (Fig. 7), while, combination of exercise with different doses of Vit D significantly increased irisin compared with non-exercised counterparts (OVX + EXE + HD vs. OVX + HD, p = 0.001, OVX + EXE + LD vs. OVX + LD p = 0.046, OVX + EXE − D vs. OVX − D, p = 0.034). No significant correlation was found between serum irisin and BDNF level (Fig. 8).

## Discussion

Findings of the present study showed increase in visceral fat, BMI, body weight, waist circumference, TG and non-HDL cholesterol after ovariectomy surgery which was intensified by Vit D insufficiency. In line with these findings, two other reports [37, 38], showed that restoration with high dose of Vit D for 8 weeks significantly attenuates body weight, BMI, visceral fat, waist circumference, serum non-HDL cholesterol and TG.

It has previously reported that estrogen withdrawal leads to the progress of metabolic syndrome components [39–41]. Estrogen has been known to play an important role in the synthesis of particular enzymes such as lipoprotein lipase (LPL), hormone-sensitive lipase (HSL) and also apolipoproteins for HDL [39]. On the other hand, Vit D induces lipoprotein lipase activity [42] and decreases fatty acid absorption in the gut and increases the conversion of cholesterol to bile acids and removes cholesterol from the circulation [43].

Also Vit D increases PPAR-α, PPAR-γ and CPT-1 (carnitine palmitoyl transferase I) expression and promotes β-oxidation and reduces TG levels [44, 45]. CPT-1 also helps FFA transport into mitochondrion for further oxidation and thus decreases lipid deposition [45]. Therefore, these findings suggest that both estrogen and Vit D play important roles in lipid metabolism.

Unlike to the metabolic improvements, Vit D had no significant change in cognitive function. This finding is consistent with the previous studies in ovariectomized [46], aged [47] and Alzheimer model of rats [48] but in contradictory with some other studies on Alzheimer [49], obese [50] and ovariectomized rats [51]. It should be noticed that the dose and duration of Vit D supplementation in our study were sufficient, because serum concentration of Vit D was significantly correlated with supplementation. However, the weakness of this study was using Vit D free diet parallel with keeping animals away from UV light to induce Vit D deficient rats. As biochemical measurements showed, this method induced Vit D insufficiency rather than deficiency, possibly due to the fact that stored Vit D in other tissues such as adipose tissue and macrophages could release Vit D into the circulation [42, 46]. Taken into account that Vit D insufficiency in OVX animals did not cause significant memory impairment, thus further Vit D restoration, didn’t show significant improvement compared to the baseline values. Although Adham et al. reported a better performance in MWM after Vit D supplementation in rats [49].

Furthermore, Vit D insufficient group displayed more BDNF concentration and Vit D supplementation reduced it to the basic level. The inverse significant correlation between Vit D and BDNF is consistent with Flavio et al. [52] study in which they reported reduction in plasma nerve growth factor (NGF) and BDNF following Vit D supplementation in healthy postmenopausal women [52]. Elevation in circulating level of BDNF in Vit D insufficiency status might reflect BDNF-mediated metabotropic compensatory effects rather than neurotrophic role in order to maintain cardio metabolic homeostasis [53, 54].

Based on our findings, although 8 weeks of aerobic exercise significantly improved dyslipidemia compared with non-exercised counterparts. Animals receiving co treatment of Vit D supplementations and exercise, showed much more improvement than their counterparts. Unlike BDNF insignificant response to exercise, irisin level was raised significantly. Irisin has been proposed to be secreted by myocytes and adipocytes during exercise [17, 55] and modulates energy expenditure [17] insulin resistance [17] and dyslipidemia [56, 57]. It appears from our data that elevation in irisin takes place in response to exercise after muscle contraction regardless of serum Vit D level.

In addition, the present study didn’t show any significant change in working and reference memory assessed by MWM after aerobic exercise in OVX groups with different status of Vit D. However study carried out by Kaidah et al. [58] showed contradictory result [58]. Inconsistency might be related to difference in exercise protocols which consisted of five times per week for 12 weeks in ovariectomized rats in their work [58].

Finding, no correlation between irisin, BDNF, and cognitive performance challenges the previous theory indicating irisin mediated exercise-induced cognitive performance [19]. Therefore, irisin cannot be an essential pathway from exercise toward central nervous system to boost learning and memory, as Timmons et al. [59] stated before [59].

In conclusion, Vit D insufficiency deteriorates metabolic syndrome components, and further supplementation significantly attenuates them parallel with reduction in BDNF. In addition, aerobic exercise successfully induces various metabolic benefits, provided optimum serum level of Vit D.
